# Indenting multicellular spheroids with various tip geometries

**DOI:** 10.1007/s00249-026-01838-3

**Published:** 2026-04-06

**Authors:** Kajangi Gnanachandran, Ewelina Lorenc, Alessandro Podestà, Małgorzata Lekka

**Affiliations:** 1 Department of Biophysical Microstructures, Institute of Nuclear Physics, Polish Academy of Sciences, Kraków, PL- 31342 Poland; 2 Vascular Biology Research Group, Department of Medical Biology, University of Tromsø, The Arctic University of Norway, Tromsø, Norway; 3 Dipartimento di Fisica Aldo Pontremoli, Università degli Studi di Milano, via G. Celoria 16, Milano, 20133 Italy; 4 CIMaINa, Università degli Studi di Milano, via G. Celoria 16, Milano, 20133 Italy

**Keywords:** Mechanical properties, Multicellular spheroids, Atomic force microscopy, Cantilever tip geometry

## Abstract

Spheroids are of great interest in the study of cancer as they can partially mimic the tumour microenvironment, thus allowing to investigate several aspects of cell – microenvironment interactions in healthy and diseased conditions, including those pertaining to mechanobiology. Atomic Force Microscopy (AFM) is a versatile tool for studying biological samples and their mechanobiological properties. In AFM, the tip shape and dimensions determine the contact geometry between the tip and the sample and the length scales at which the mechanical properties are probed. Given the complex multiscale structure of spheroids, the choice of tip geometry and size would allow, in principle, to dissect the mechanical response of the overall system into the contributions of the constituents, from the single cell level to the cellular aggregate. In this work, we studied the mechanical properties of spheroids derived from four cell lines (A549, NHLF, HT-29, and CCD-18Co cells). Our studies revealed that using different contact geometries in the fitting procedure results in significantly different Young’s modulus values, highlighting the multiscale response of these complex cellular systems and the importance of a precise experimental design and choice of the AFM probe for the nanomechanical measurements. We observed that the location of F-actin filaments is correlated with the rigidity of the spheroids.

**Supplementary Information:**

The online version contains supplementary material available at 10.1007/s00249-026-01838-3.

## Introduction

In recent years, multicellular spheroids have been widely used as a good in vitro model in various types of research, especially in drug efficacy and toxicity trials, but also in the investigation of organ development and congenital diseases, tissue engineering, and 3D bioprinting (Jensen and Teng 2020; Habanjar et al. 2021; Kim et al. 2023). Spheroids are now the most desirable 3D model for creating uniform, repeatable multicellular structures and a potential starting point for building big tissues and intricate organs (Pinto et al. 2020). The spheroids are of big interest in the study of cancer as they can partially mimic the tumour microenvironment, allowing a better recapitulation of the patient tumour, which represents a promising challenge to improve the success rates in anticancer drug development and study of cell-extracellular matrix (ECM) interaction, cancer biology, including cancer initiation, invasion, and metastatic processes (Pinto et al. 2020; Zanoni et al. 2020). In 2D, cells are isolated on a rigid flat substrate, and cell mechanics predominantly reflect cortical stiffness. In contrast, 3D spheroids integrate cell–cell contacts, extracellular matrix deposition, and multicellular interactions, producing multiscale mechanical responses that better reflect tissue-level mechanics and tumor microenvironmental conditions. Consequently, even if 2D and 3D stiffness values numerically overlap, 3D spheroids provide biologically relevant mechanobiological insights that cannot be obtained from 2D cultures, including modulation of cytoskeletal organization, collective mechano-transduction, and spatial mechanical heterogeneity.

Cancer cells are known to be subjected to variable stresses during cancer progression due to the fast-dividing process in a restricted space (Butcher et al. 2009). Likewise, several mechanical events take place during tumour progression. For instance, stromal myofibroblasts overproduce collagen, which stiffens the matrix (Shao et al. 2000); the tumour mass expansion induces compression (Helmlinger et al. 1997), and because of the poor lymphatic drainage, there is a rise in the interstitial pressure (Boucher and Jain 1992). Several studies have shown that spheroids are a potentially powerful model to study the influence of mechanical stress on tumour growth (Delarue et al. 2014; Dolega et al. 2021; Mahajan et al. 2021).

Since it has been demonstrated that the mechanical properties of cancerous cells and the organisation of their cytoskeletal networks are altered compared to those of normal cells (Makale 2007; Hall 2009), the mechanobiology of cells has become an important area of cancer research alongside traditional genetic and biochemical studies. There is a broad selection of tools for studying the mechanical properties of cells, including Atomic Force Microscopy (AFM), micropipette aspiration, optical and magnetic tweezers, and different microfluidic approaches (Eroles and Rico 2023). AFM is a powerful tool that, thanks to its versatility, has been used to study a variety of biological samples and different mechanobiological aspects (Krieg et al. 2019), by characterizing their viscoelastic (Lekka et al. 1999; Gnanachandran et al. 2022; Pérez-Domínguez et al. 2023) and adhesive properties (Makarova et al. 2023). However, only recently, the mechanical properties of spheroids have become of interest in AFM studies due to their complex and multilayer structure (Guillaume et al. 2019; Giannetti et al. 2020; Fuhs et al. 2022).

The key elements of the AFM are the cantilever and the probing tip(Lorenc et al. 2023). Since different tip geometries are appropriate for various uses and sample types, the study objectives and sample properties will ultimately define which tip geometries should be used. The tip interacts directly with the sample, and its shape determines the contact geometry between the two. Contact mechanics relate the load force F and the indentation δ (Kontomaris et al. 2020) (Lacaria et al. 2023). The obtained equations take into account the geometry of the indenter, i.e., the probing tip shape (Table 1) (Kontomaris et al. 2022).

Pyramidal tips are the preferred choice for experiments that aim to obtain high-resolution images of scanned surfaces or to create force maps (Viji Babu et al. 2019). The use of pyramidal tips allows for deep indentation studies and for imaging of small features of the sample. Unfortunately, in the case of studies involving biological samples, sharp tips are a source of significant stress (Rico et al. 2005; Pérez-Domínguez et al. 2023). Colloidal probes (CP), with radii between 2 and 20 μm, represent an alternative to study the mechanical properties of biological samples (Rico et al. 2005; Puricelli et al. 2015; Chighizola et al. 2021). First of all, CPs do not cause high stress even for high indentations, which can, therefore, be higher than in the case of pyramidal tips. The cost of using CP is lower lateral resolution; however, this issue can be overcome by using CP of a smaller diameter (Rico et al. 2005). The increased contact area between the sphere and sample allows for detecting - weak interaction forces, which is another characteristic of CPs. In general, CPs make it easier to interpret data regarding theoretical models (Puricelli et al. 2015; Chighizola et al. 2021). To perform measurements with a constant and defined area, cylindrical tips can be used (Rico et al. 2007; Waters et al. 2012). Because they exhibit a linear force-indentation relationship, cylindrical tips find multiple applications in studies of cell adhesion and the nonlinear response of cells (Lacaria et al. 2023).

Several studies have already reported the effects of different types of cantilevers on the elastic properties of single cells. These studies have demonstrated that the measured value of Young’s modulus (YM) of elasticity depends on the cantilever type and on the contact mechanics models which rely on several approximations (Rico et al. 2005; Finot et al. 2008; Zemła et al. 2020). These are among the main reasons for the variability of the published results (Lekka et al. 2012). Most of them were conducted in 2D cell cultures and there is no data showing analogous effects of tip geometry on the mechanical properties of 3D systems such as spheroids. To fill the gap, we performed AFM-based elasticity measurements on spheroids formed from four distinct cell lines characterised by large variations in the organisation of their actin cytoskeleton, which is either well- or poorly differentiated. To obtain the mechanical responses from the spheroids at different scales, we used four types of cantilevers with tips of varying sizes and geometries. Our results indicate that regardless of the tip used, spheroids with similar actin cytoskeleton organisation do not display any mechanical differences, indicating that even at the spheroid level, under the experimental conditions used, the mechanical properties of cells can be related to their cytoskeletal organisation.

## Materials and methods

### Cell culture and spheroid formation

In this research, four different cell lines were used: A549 (human lung carcinoma, Lonza) and NHLF (human lung fibroblasts, ATCC), HT-29 (human colon adenocarcinoma, a kind gift from the Chair of Medical Biochemistry, Collegium Medicum Jagiellonian University, Cracow, Poland), and CCD18-Co (human colon fibroblasts, a kind gift from the Department of Virology and Immunology, University of Maria Sklodowska-Curie, Lublin, Poland). NHLF cells were cultured in Fibroblast basal medium (FBM) + Fibroblast growth medium − 2 (FGM-2), while A549 cells were cultured in F12K medium. Both culture media were supplemented with 10% Fetal bovine serum (FBS). HT-29 and CCD18-Co cells were cultured in RPMI 1640 and Advanced DMEM medium, supplemented with 5% FBS, 1% penicillin/streptomycin, and 1% amphotericin. The lung cell lines were grown in culture flasks (Sarstedt) in an incubator (Nuaire) at 37° C with 5% CO_2_. HT-29 and CCD18-Co cells were grown in the same conditions in the incubator (Galaxy S, RS biotech). Passaging was carried out when the cells reached 80–90% confluency. A 0.25% trypsin-EDTA solution was used to detach all cell lines from the culture flask surface. If not stated otherwise, all bioreagents and materials came from Sigma Aldrich. Once the cells were ready, the spheroids were cultured in low-attachment U-bottom 96-well plates (Thermo Fisher). To obtain spheroids of approximately 350–400 μm in diameter, adequate numbers of cells per well were selected. These were 1500 cells/well for A549 lung cancer cells, 1800 cells/well for NHLF lung fibroblasts, 1000 cells/well for HT-29 colon cancer cells, and 10,000/well for CCD-18Co colon fibroblasts. After 3 days of culture, the diameter of spheroids was measured from bright-field images using ImageJ software.

### Confocal microscopy

Confocal images of actin filaments inside spheroids were collected using a confocal microscope, available in the Laboratory *of in vivo* and in vitro imaging (Maj Institute of Pharmacology, Polish Academy of Sciences, Cracow, Poland). The samples were stained using the following protocol. Spheroids formed by each cell line were collected in a 1.5 ml Eppendorf and fixed with 3.7% paraformaldehyde for 1 h. The samples were washed with phosphate-buffered saline (PBS) for 2 min three times, treated with 1% cold Triton X-100 overnight at 4 °C, and washed again with PBS. Then, a 1% cold Bovine Serum Albumin (BSA, Sigma-Aldrich) solution was added for 3 h at 4 °C, and after removing BSA, the spheroids were washed with PBS. Next, the spheroids were incubated overnight at 4 °C with phalloidin conjugated with Alexa Fluor 488 (1:20 in PBS). The next day, the dye for actin filaments was removed, and Hoechst 33,342 (Invitrogen, dissolved in a PBS buffer (1:5)) staining the cell nuclei was added. Finally, the samples were washed with PBS and transferred to 18-well glass-bottom slides (Ibidi) with an anti-shading solution (Thermofisher) with a refractive index (1.52) matching that of the immersion oil used. Images were recorded using a Leica TCS SP8 WLL confocal microscope equipped with new-generation HyD detectors. Fluorescent dyes were excited by a diode laser at 405 nm (Hoechst) and a white light laser with an emission wavelength set to 499 nm (Alexa Fluor 488). Images were registered using an oil-immersion 63x objective lens (HC PL APO CS2 NA 1.40).

### Preparation and fixation of spheroids for AFM measurements

To keep the spheroids immobile during the AFM measurements, the Petri dishes were coated with 0.01% poly-L-lysine and 5% glutaraldehyde [35] with an additional support of a Micromesh array (Microsurfaces Pty Ltd, Australia). This was fixed on the Petri dish following the producers’ instructions, and the dish was then filled with adequate cell culture medium without phenol red. Spheroids were transferred from a multi-well culture plate to the array, placing each spheroid in a well, to make measurements easier. Placing all the spheroids under AFM setup (outside culture conditions) for the duration of the measurements (several hours) could affect spheroid properties and the quality of measurements. Considering this, only a few spheroids were collected at a time, and after measuring 3–4, new spheroids were collected to continue measurements.

### AFM tip geometries

Four cantilever types with different tip geometries were used to study the mechanical properties of spheroids (Table 1). Two types of triangular cantilevers with spherical tips (CP5 and CP10, with radii of 5 and 10 μm, respectively), triangular cantilevers (MLCT-F) with a four-sided pyramidal tip (open-angle of 20° and radius 20–40 nm), and rectangular tipless cantilevers (Arrow TL1). For the MLCT cantilevers, the tip height varied between 3 μm and 8 μm with a mean of 5.5 μm (according to manufacturer data). Prior to each experiment, the spring constants were calibrated with the thermal noise methods (Schillers et al. 2017; Chighizola et al. 2021, 2023). The radii of the CPs were either pre-calibrated by the manufacturer or calibrated according to the procedures described by Indrieri et al. (Indrieri et al. 2011). The radii of the spheroids were determined using an optical microscope.

In Table 1, the details about the various tips and their maximum indentation levels are reported.

**Table 1:** AFM tips used for experiments and measurement parameters

	Force map size [µm x µm]	Force volume [*n*. FCs]	Spring constant (*N*/m)	Cantilever model	Tip radius[µm]/half angle	Max. indentation [µm]
PROBE						
Small CP (CP5)	35 × 35	100	0.85 – 0.1	MLCT-SPH (Bruker)	4,3	~ 3 - 5
Large CP (CP10)	35 × 35	64	0.23 – 0.32	CP-PNPS-BSG-C-5 (Nano and More)	10	~ 4 - 6
Pyramidal (PYR)	20 × 20	100	0.79 – 0.6	MLCT-F (Bruker)	0.02/20°	~ 2 - 4
Tipless (TL)	40 × 40	49	0.11 – 0.03	Arrow TL1 (Nano World)	150 (average spheroid radius)	~ 4 - 7

### AFM measurements

For spheroids formed by human colon HT-29 and CCD-18Co cells, we used a Bioscope Catalyst AFM (Bruker). A Nanowizard IV AFM (Bruker – JPK Instruments) was used to measure the spheroids formed by the A549 and NHLF human lung cells. The instruments were mounted on top of an inverted optical microscope (Olympus IX71). To isolate the sample from the ground and ambient noise, the microscope was placed on an active anti-vibration base (DVIA-T45, Daeil Systems) inside an acoustic enclosure (Schaefer).

Spheroids were measured at room temperature (RT, around 24 °C) in their respective culture medium without phenol red. Working at RT is an acceptable compromise to ensure instrument and cell stability, as long as comparative studies are performed. Published literature shows that temperature can influence cell mechanics through cytoskeletal activity and contractile prestress (Sunyer et al. 2009; Chiou et al. 2013; Sunnerberg et al. 2019). Within the near physiological range (≈ 31–37 °C), differences in stiffness are generally moderate, and relative trends between cell types are preserved. This supports the validity of our comparative measurements at RT, while we acknowledge that absolute modulus values would likely differ slightly at 37 °C.

The medium was changed several times to prevent heating it from the microscope lamp. In all measurements, the approach speed was set to 10 μm/s, and the maximum load force was kept at 15 nN. Two different indentation levels with the pyramidal tip were obtained by applying load forces of 5 nN and 15 nN, corresponding to low and high indentation, respectively. For each set of measurements, 10 spheroids were measured, and 3–6 force maps were collected per spheroid. A suitable scan area was selected based on the contact areas of the different cantilevers, as indicated in Table 1.

### Young’s modulus determination and statistics

**Fig. 1:**
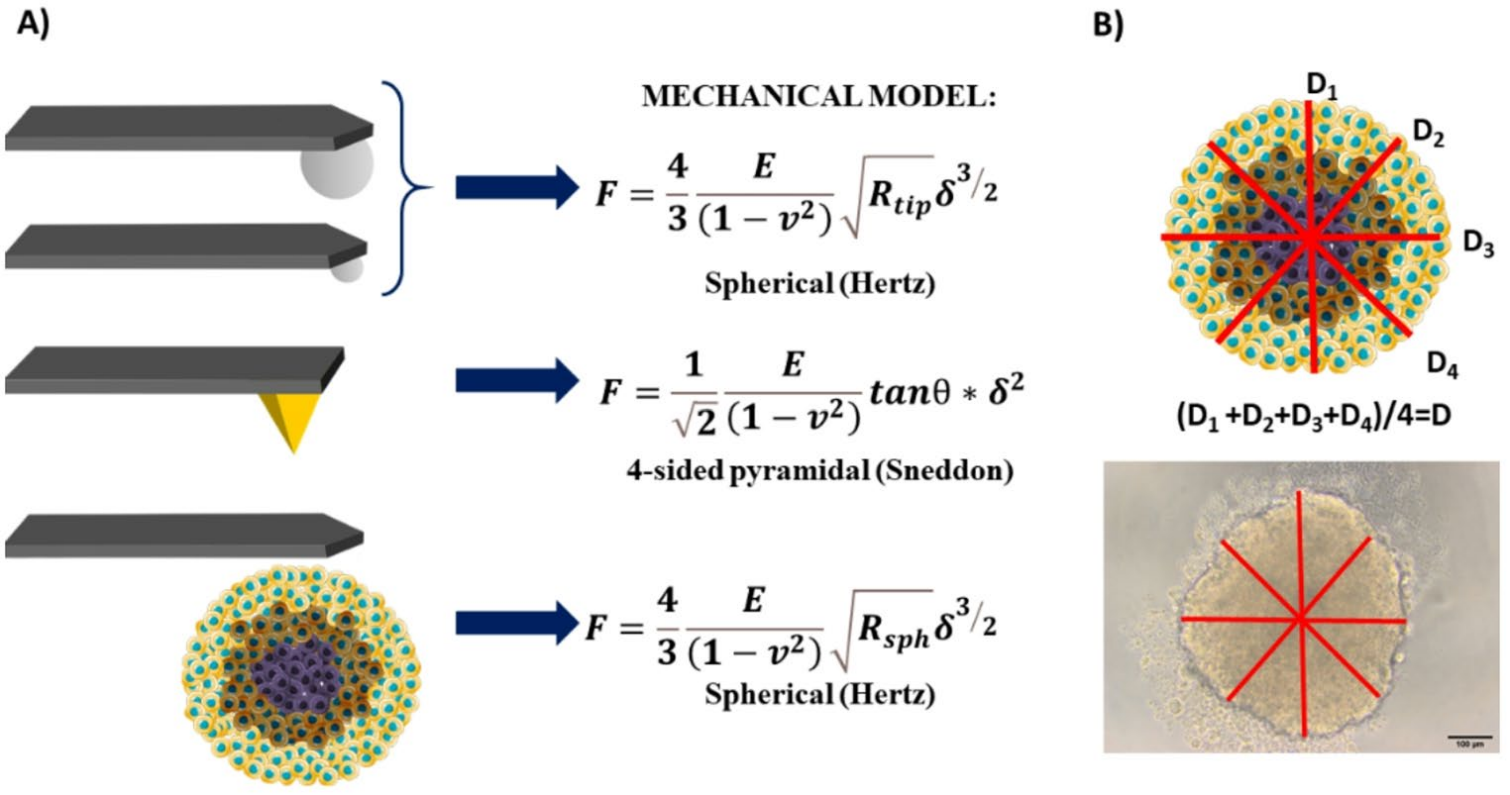
**A**) Summary of the selected contact mechanics models for the AFM measurements on spheroids performed with various tip geometries (*R*_*tip*_ - radius of the beads; *R*_*sph*_ - average radius of spheroids; *E* - Young’s modulus; ν – Poisson ratio, assumed to be 0.5; δ – indentation; θ – opening half angle of the pyramidal tip, assumed to be 35°). **B**) Determination of the spheroid diameter

The data obtained from spheroids formed by A549 and NHLF cells were processed with the software provided by JPK. The data obtained for spheroids derived from the other colon cell types were analysed by using custom MATLAB scripts following the routine described in Puricelli et al. (Puricelli et al. 2015). In both cases, the rescaled force-indentation curves (shortly force curves, FCs) were fitted using the appropriate contact mechanics models (Fig. 1) to obtain the values of Young’s modulus (Butt et al. 2005). The Hertz model for the paraboloidal indenter was used as an approximation of the model for the spherical indenter (Lacaria et al. 2023).

In the case of measurements with tipless cantilevers, the Hertz model for spherical probes was applied, assuming the indentation of a soft spherical body (the spheroid) by a flat and stiff plate (the tipless cantilever). As the radius of the spherical body, we took the average radius of the spheroids (approximately 150 μm). Additionally, we performed trial data analysis using two additional models. The first is the flat punch model (1) (Rico et al. 2007), assuming the cantilever width *a* as the diameter of the cylinder (therefore the punch radius is a’=a/2):

1 $$\:F=\frac{2E}{1-{v}^{2}}a{\prime\:}\delta\:$$

The other model is the Hertz model for colloidal probes, in which we assume the spheroid is locally flat and the tipless cantilever is an effective spherical indenter with a diameter equal to the cantilever width.

The statistical significance was calculated using an ordinary ANOVA test with Turkey’s multiple comparison test. Error bars represent the 95% confidence interval of the median.

It should be noted that, considering the non-negligible indentations, the measured Young’s modulus must be considered as effectively recapitulating the combined elastic contributions of different cellular components (cytoskeleton, including cortical actin, cytoplasm, nucleus,…) as well as cell-cell interactions. Depending on the specific experimental condition (typically, the specific probe used), one component (such as cortical actin) may contribute predominantly relative to others.

## Results and discussion

### Organization of F-actin inside the spheroids formed by human lung and colon cells

**Fig. 2:**
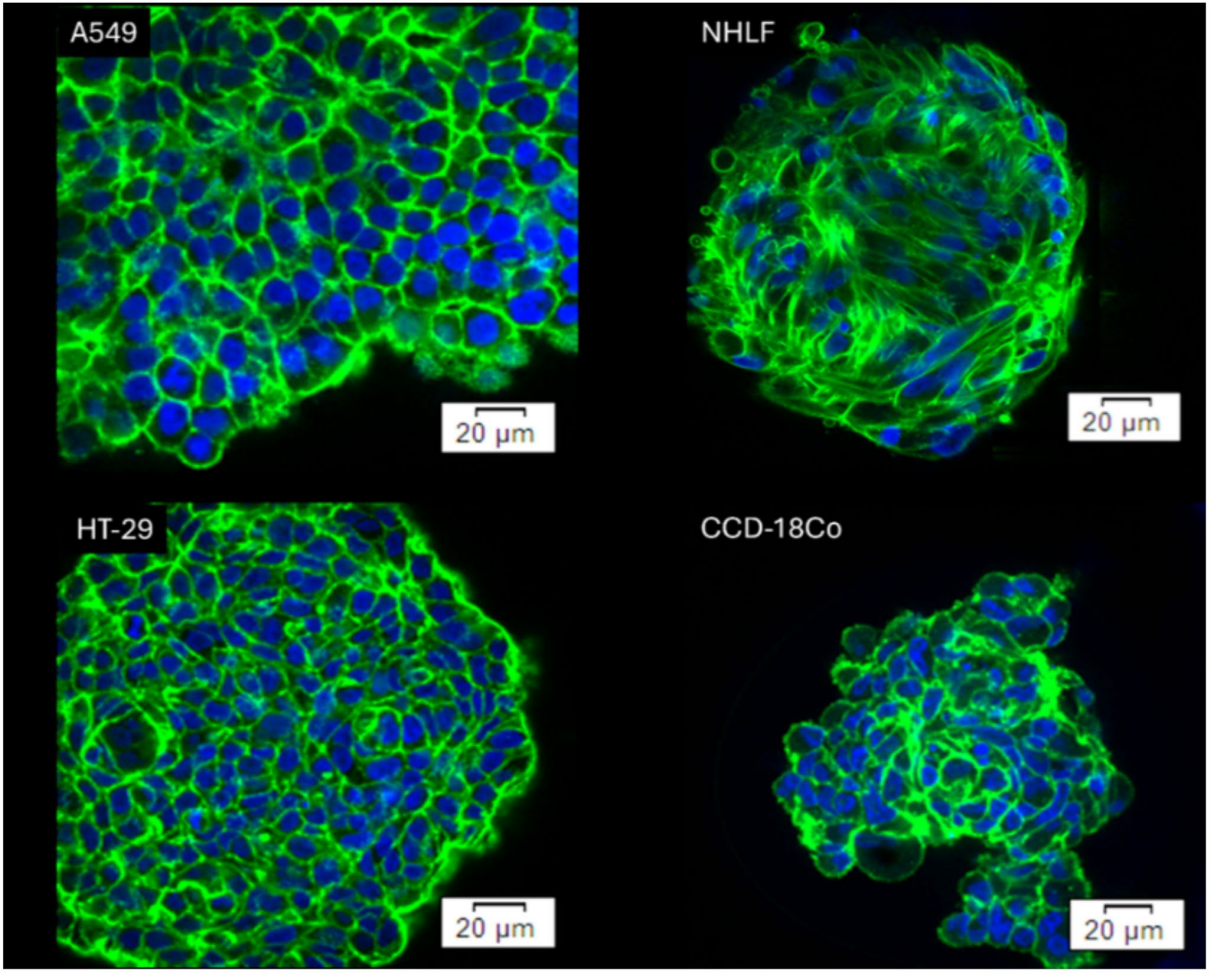
Confocal images of spheroids, formed by (**A**) lung cancer cells (A549 cell line), (**B**) healthy lung fibroblasts (NHLF cell line), (**C**) colon cancer (HT-29 cell line), and (**D**) colon fibroblasts (CCD-18Co cell line) [one slice from the Z-stack is shown]. Blue: nucleus (Hoechst 33342), Green: actin filaments (phalloidin Alexa Fluor 488)

The mechanical properties of cells are predominantly related to the organization of the actin cytoskeleton, not only at the single-cell level (Lekka 2016) but also at the spheroid level (Gnanachandran et al. 2022). Therefore, in this study, we first performed confocal imaging of the spheroids formed by the healthy lung fibroblasts (NHLF), colon fibroblasts (CCD18-Co), the small-lung cancer cells (A549), and colon cancer cells (HT-29), which were stained with Hoechst 33,342 and Alexa Fluor 488 to observe the cell nucleus and the organization of actin filaments inside spheroids, respectively. Figure 2 shows the confocal images of all the spheroids.

In spheroids formed by lung cancer A549 cells, actin filaments are organized around the cells. Compared to A549 spheroids, those formed by the healthy lung fibroblasts (NHLF) present thick actin bundles distributed around and over the cells. The confocal images show a dissimilarity in the shapes of individual cells, i.e., more rounded A549 cells and more elongated NHLF fibroblasts. The spheroids formed by colon epithelial (HT-29 cells) and colon fibroblasts (CCD-18Co cells) display an actin filament organization similar to that present in the spheroids formed by A549 cells, with actin filaments mainly distributed around the spherical cells.

To sum up, colon spheroids consist mainly of round cells with F-actin distributed around the cells. In contrast, lung spheroids are composed of spindle-like fibroblasts with actin filaments spanning the whole cell or round, epithelial cells with F-actin organisation similar to that observed in colon spheroids.

### Mechanical properties of spheroids formed by human lung and colon cells

The influence of cantilever tip geometry on AFM nanomechanical measurements has already been investigated on individual cells cultured on flat 2D surfaces. Published AFM measurements in 2D cultures (using both pyramidal and spherical tips) provided us with representative Young’s modulus (YM) values for the cell lines studied in our work. These values allowed us to contextualize our 3D spheroid measurements (see(Xiao et al. 2013; Zhang et al. 2018; Zhu et al. 2021) for A549 cells; see(Brás et al. 2022; Efremov et al. 2023) for HT 29 cells; see(Beton and Brożek-Płuska 2022) for CCD 18Co cells; see(Rodriguez et al. 2013) for NIH/3T3 cells). Here, we take a step forward by performing a similar study at the 3D level. Therefore, this will allow us to understand the mechanical properties in a biological model, that is closer to the actual tumour microenvironment.

**Fig. 3:**
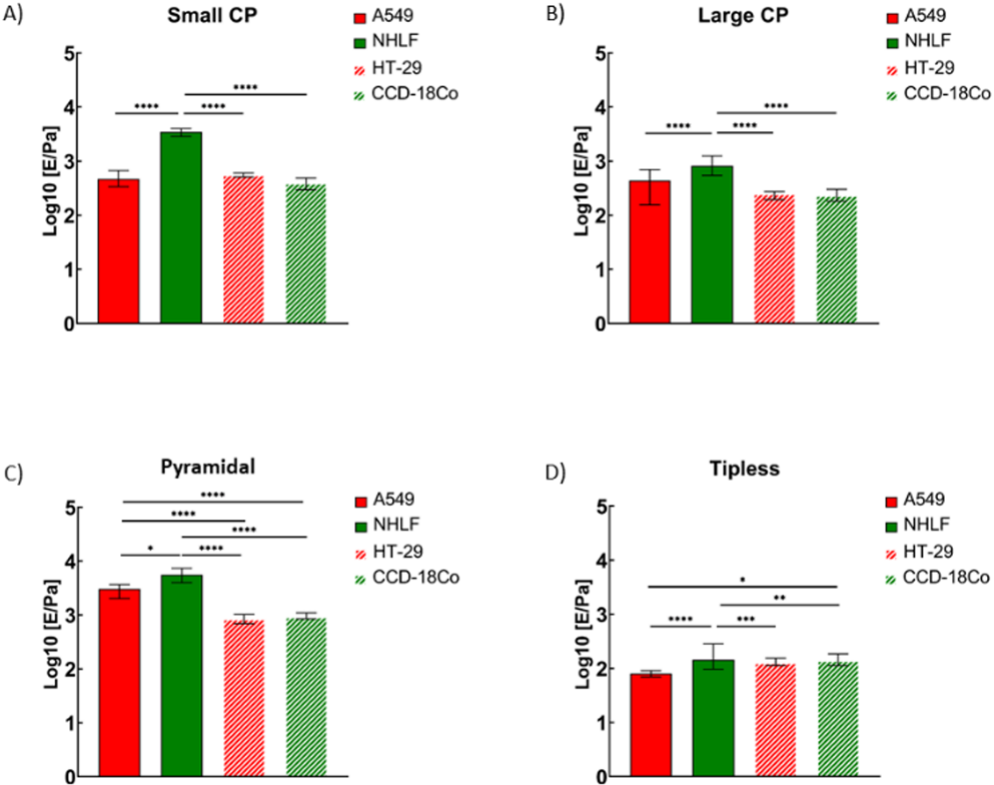
Young’s modulus obtained for spheroids formed from lung (A549 and NHLF) and colon (HT- 29 and CCD18-Co) cells. Error bars represent the 95% confidence interval of the median. The results have been obtained using: **A**) cantilevers with spherical tips of radius *R* = 4.3 μm (CP5) and indentations δ = 2–3 μm; **B**) cantilevers with spherical tips of radius *R* = 10 μm (CP10)and indentations δ = 4–6 μm; **C**) pyramidal MLCT tips (typical apical radius *R*=20 nm) and indentation δ = 2–4 μm; **D**) tipless cantilevers (TL) and indentations δ = 4–6 μm (**** p < 0.0001, *ns* - not statistically significant)

The mechanical results for the spheroids formed by A549 and NHLF lung cells as well as by HT-29 and CCD18Co colon cells are reported in Fig. 3.

### Colloidal probes

The mechanical properties of lung and colon spheroids were first measured using two distinct colloidal probes (CP5 and CP10) to probe the mechanical response of spheroids on different scales **(**Fig. 3A-B). In our measurements, the indentation achieved allowed us to study the outermost layers of the spheroids, i.e., the first few layers of cells and the ECM produced by the cells.

The YM, obtained from spheroids formed from A459 lung cancer cells, was independent of the spherical probes used in the measurements. Its value remained similar, i.e., 500 ± 30 Pa (*n* = 39 maps) versus 450 ± 60 Pa (*n* = 31 maps), for spherical probes of 5 μm and 10 μm radius, respectively. A different situation is observed for spheroids formed from NHLF lung fibroblasts. Here, YM was 3600 ± 270 Pa (*n* = 30 maps) versus 950 ± 90 Pa (*n* = 33 maps) for spherical probes of 5 μm and 10 μm radius, respectively.

Spheroids derived from colon cell lines were softer than those from lung cell lines. YM values measured with a spherical probe of radius 5 μm were 580 ± 30 Pa (*n* = 61) for HT-29 spheroids and 490 ± 80 Pa (*n* = 57) for CCD-18Co spheroids. With a larger sphere, obtained values for HT-29 and CCD-18Co spheroids were 310 ± 30 Pa (*n* = 59) and 310 ± 50 Pa (*n* = 50), respectively. Both results indicate that in the case of colon cells, there is no difference in elastic properties between spheroids derived from cancerous cells and those derived from fibroblasts.

### Pyramidal tips

The mechanical properties of spheroids were then measured with pyramidal tips (Fig. 3C). YM of lung cancer cells and fibroblasts was measured at two different indentation depths (low indentation = 1 μm; high indentation = 2–3 μm), which were obtained by adjusting the load force to 5 nN and 15 nN, respectively. They were smaller than the values obtained with the colloidal probes. Two different indentation ranges for HT-29 and CCD-18Co were extracted from two fitting ranges: 10%-30% and 30%-90% of linearized FCs. A comparison of these ranges revealed no significant differences in YM (**Supplementary Figure S1**), suggesting a relatively uniform mechanical response across these depths for the colon-derived spheroids. Although the absence of a multimodal distribution in this specific spheroid types does not definitely exclude mechanical contributions from specific components such as the cytoplasm, the nucleus, or from intra-cellular space mimicking ECM/spheroids microenvironment, we assume that a specific nuclear or ECM contribution can be neglected, and we interpret the measured Young’s modulus as an effective elastic modulus describing the overall local elastic response. This comparative analysis was restricted only to the colon-derived spheroids, thus the interpretation of the results may not directly apply to the lung-derived spheroids, which exhibit different mechanical properties and structure. To ensure a consistent representation of the data across all spheroids models, only the results from the high indentation range are presented in the main analysis.

Figure 3C shows that for each cell line, YM remained the same, regardless of the indentation depths considered. It suggests that the probed spheroid layer remained mechanically homogenous. On the other hand, the lack of differences can be explained by the fact that the thickness of the probed layer is not very high, as the height of the pyramidal tip limits it. Moreover, the spheroid radius is much larger than the maximum indentation allowed by the standard AFM setup; therefore, our measurement is surface-sensitive rather than bulk-sensitive, which is clearly different from the typical single-cell nanomechanical measurements. Moreover, as expected from the literature data (Rico et al. 2005; Zemła et al. 2020), YM measured with sharp pyramidal tips is typically higher than that measured using colloidal probes. This difference is clearly seen in the case of lung spheroids.

Spheroids formed from healthy fibroblasts (NHLF cells) were significantly stiffer than those from lung cancer (A549 cells). The corresponding YM was 7600 ± 1350 Pa (*n* = 30 maps) versus 3300 ± 360 Pa (*n* = 36 maps) and 6900 ± 580 Pa (*n* = 30 maps) versus 3700 ± 510 Pa (*n* = 29 maps) for low and high indentations.

The same observation regarding the independence of the measured YM value on the indentation depth was also made for the spheroids obtained from two colon cell lines (**Supplementary Figure S2**). Additionally, we did not observe any significant difference in rigidity between HT-29 and CCD18Co spheroids in both indentation ranges. Here, we measured 810 ± 70 Pa (*n* = 50 maps) for HT-29 spheroids and 760 ± 60 Pa (*n* = 51 maps) for CCD-18Co spheroids in low indentation range; in large indentation scale, the YM values were 900 ± 70 Pa (*n* = 50 maps) and 1050 ± 80 Pa (*n* = 51 maps) for HT-29 and CCD18-Co spheroids, respectively. Both results indicate that in colon spheroids, there is no difference in elastic properties between spheroids derived from cancerous cells and fibroblasts, which may be related to the absence of difference between their actin cytoskeleton organisations (see Sect.  3.1). In addition, this observation could reflect properties that go beyond the single cell level, related to spheroid multicellular architecture, cell–cell interactions, and probe-averaging effects, especially in the larger-indentation measurements.

### Tipless cantilevers

Finally, the elastic modulus of the spheroids was measured using a tipless cantilever (Fig. 3D). The great advantage of tipless cantilevers is the large contact area due to the width of the cantilever (up to 100 μm) and its flat surface. Tipless cantilevers allow the indent of a spheroid as a whole rather than locally at the single- or few-cell level.

Using tipless cantilevers for measurements on spheroids is an interesting approach; however, it raises questions regarding the contact mechanics model that should be applied. To verify which model is most suitable, data from three spheroids were analysed to see which approach would be more suitable for further analysis (**Supplementary Figure S3**). Three different models and assumptions were used:

- the Hertz model for CPs with R equal to the radius of the spheroid, which is equivalent to treating the spheroid as a deformable sphere indented by a rigid flat plate (the cantilever);
- the Hertz model for CPs with R equal to half the width of the cantilever, which considers the tipless cantilever as equivalent to a very large spherical tip;
- the Hertz model for a flat cylindrical probe, with the radius a’ of the circular section taken equal to half the width a of the cantilever, which assumes that the contact area is nearly constant.

For the first model, we used the average radius of spheroids measured that day, as shown in Fig. 1. For the second model, we assumed that the cantilever could be treated as a sphere with an effective diameter equal to its width. For the third model (the flat punch model), we assumed for the radius of the flat contact region *a’* = 50 μm. In fact, the tipless cantilever does not provide a constant contact area, unlike a truly cylindrical flat tip. The contact region of the tipless cantilever can be approximated by an ellipse, with one axis determined by cantilever width and the other axis expected to increase with indentation. As a result, the data analysed using the flat punch approximation yielded lower Young’s modulus values than those from other models (**Supplementary Figure S3**).

Giannetti et al. 2020 performed a similar study on spheroids derived from T24 transitional cell carcinoma cells. The obtained elastic modulus was in the range of 100–500 Pa. Elastic modulus measured in this research (using the Hertz model with the radius of the spheroid, HertzSPH) were quite similar: 158 Pa for CCD-18Co and 213 Pa for HT-29 spheroids, while results obtained using the cylindrical model were lower than reported in Giannetti et al. 2020 and for Hertz model used in these studies (Giannetti et al. 2020). This confirms that using a cylindrical tip is not optimal while indenting with a tipless cantilever. Considering the results of data analysis, the most suitable and reliable model for further studies is the Hertz model for CPs, with a radius equal to the radius of spheroids, i.e. treating the spheroid as a deformable spherical object pushed against a flat wall (the cantilever). This model more accurately represents the experimental geometry, accounting for the increase in contact area with indentation, and produces modulus values consistent with prior AFM studies on spheroids and organoids. In contrast, the flat punch model underestimates Young’s modulus because it ignores the increase in contact area with indentation (this model would probably be more accurate for much wider cantilevers). The effective spherical cantilever model tends to overestimate the modulus, probably because the geometrical approximation is far too crude. Therefore, using the Hertz sphere‑plane approach provides a physically realistic and reproducible measure of the bulk mechanical properties of spheroids (which would likely work better for even wider cantilevers), while also allowing comparisons across different cantilever geometries.

The most important observation is that the values of YM are much smaller than the modulus values when pyramidal and spherical probes are considered (Fig. 3). The moduli ranged from 80 ± 6 Pa (*n* = 40 maps) to 250 ± 40 Pa (*n* = 30 maps) for spheroids formed respectively by A549 cells and NHLF fibroblasts. Comparison between the two colon cell lines revealed no differences, as mean values were 140 ± 10 Pa (*n* = 51) for HT-29 spheroids and 160 ± 10 Pa (*n* = 51) for CCD-18Co spheroids.

As described in the method section, we analysed the data for the tipless cantilevers using the Hertz model for the sphere-plane contact geometry with the average radius of the spheroids (approximately 150 μm) as the radius *R* in Eq. 1 and treating the tipless cantilever as a rigid flat surface. The other methods tested to simulate an effective sphere on flat geometry did provide similar results (see **Supplementary Figure S3**); nevertheless, since these methods are based on rather crude geometrical assumptions, we decided not to consider those results in the discussion.

Overall, our results show that the lung spheroids formed by NHLF cells have a higher value of YM than those formed by the cancerous A549 cells, regardless of the type of AFM probe. However, the magnitude of the changes was related to the probing geometry, and the most significant difference was observed for spherical probes at large 4–5 micron indentations. On the other hand, AFM measurements with different probes did not reveal any differences between colon cell lines studied here (Fig. 3 and **Supplementary Figure S4**).

The analysis of tumour-derived spheroids from the A549 and HT-29 cell lines revealed that the outcomes of the measurements varied across scales. The outcome of measurements with the MLCT tip was that A549-derived spheroids are stiffer than HT-29 spheroids, while measurements with a tipless cantilever showed the opposite results (**Supplementary Figure S5 A-C**). AFM measurements of soft biological samples depend strongly on probe geometry and contact area. Sharp pyramidal tips typically yield higher apparent Young’s moduli by probing local surface features, such as the actin cortex. In contrast, larger or flat tips average across multiple cells and the ECM, yielding lower bulk values. Computational models confirm that apparent stiffness can vary substantially with tip shape and indentation depth (Yang et al. 2021). We think that, the opposite trends observed for A549 and HT 29 spheroids probed with different tips may arise from these scale-dependent effects: pyramidal tips probe only outer cells in the spheroid, reflecting local actin organization, while tipless cantilevers deform larger volumes, sensing bulk mechanics. Thus, A549 spheroids appear stiffer at the surface but softer in bulk, whereas HT 29 spheroids show slightly stiffer bulk mechanics despite softer outer layers. In the case of spheroids made of the fibroblast, NHLF spheroids were significantly stiffer, however, in measurements with tipless cantilever, these differences were more pronounced (**Supplementary Figure S5 D-F**).

It is well known that cells can sense the rigidity of the microenvironment, which affects their morphology and behaviour (Lo et al. 2000; Yeung et al. 2005; Smith et al. 2006). This is why using spheroids as a study model is more advantageous than standard 2D culture. The cell’s response to the rigidity of the surrounding medium may vary between different cell types (Yeung et al. 2005). This might be linked to the organisation of the actin in the cells. We observed the difference in the organisation of actin fibers between lung-derived spheroids of A549 and NHLF cell lines, and later we measured different values of YM.

The differences in the rigidity were observed in all measurements, using different geometries of the probes. By comparing the colon-derived HT-29 and CCD-18Co spheroids, we observed no difference in the actin organisation. The cells in CCD-18Co spheroids showed a similar morphology to cells in HT-29 spheroids. This aligns with AFM nanoindentation results, which showed no differences in rigidity. Comparing lung fibroblasts with colon fibroblasts showed that the former are stiffer (**Figure S5 D-F**), which correlates with the information that a well-organised actin cytoskeleton increases the elastic modulus (Blumlein et al. 2017).

Many AFM studies show that individual cancer cells tend to be softer (lower Young’s modulus) than their normal counterparts, and that this is related to cytoskeletal remodeling and reduced stress fiber formation. For example, comparative AFM measurements across breast, lung, and cervical cancer lines showed that cancer cells were systematically softer than matched normal cells; reviews of cell mechanics also conclude that cancer cells often display increased deformability due to actin disorganization (Gavara and Chadwick 2016; Grady et al. 2016). In lung spheroids, this behaviour is confirmed, i.e. a different actin organisation is associated with the different physiopathological state (cancerous vs. normal, and consequently, we observe a change in rigidity). In colon spheroids, this is not observed (and consequently, we do not observe a change in rigidity). It seems therefore that not always a change in rigidity is associated to a different physiopathological state. A possible explanation is that our spheroids are 3D systems; cells feel the overall rigidity of the microenvironment, to which they are very sensitive, and the cell microenvironment, in spheroids, are cells themselves. Since colon spheroids tend to be softer, actin remodeling in cells is not triggered, irrespective whether they are normal or cancerous.

## Conclusions

Using various geometries of AFM tips allowed us to study the mechanical properties of spheroids at different length scales, from within the single cell to the multicellular level.

In our mechanical analysis, we neglected time-dependent viscoelastic and/or poroelastic effects and adopted a purely elastic, static Hertzian approach. This is clearly a simplification, which leads us to consider the measured Young’s moduli as effective, protocol-dependent quantities rather than the intrinsic elastic constants of the system under investigation.

It is known that the measured YM may depend on tip geometry, which is related to the contact area and the generated stress (Zemła et al. 2020; Kulkarni et al. 2023). We indeed observed that the measured YM values were higher for sharper (pyramidal) tips across all 4 types of spheroids. However, we observed the same relative trend among the different spheroids, irrespective of the probe used, especially when a larger and more regular contact area was used, i.e., for CPs and the tipless cantilever.

It has been observed before that cells in 3D can have different rigidity depending on their location. In the work of Vyas et al. the YM of cells on the surface of the spheroid was higher than in the inner part of the proliferation zone (Vyas et al. 2019). Our results highlight the importance of selecting the appropriate probe for studying the mechanical characteristics of biological samples, knowing that each component has a different YM value. The right choice of the AFM probe will depend on what information we want to extract: the average value of YM that includes the contribution of all sample elements, or the rigidity of a single element within the sample. When measuring large and mechanically heterogeneous samples, the tip shape of the AFM probes influences the estimation of Young’s moduli; tips with larger and more regular contact areas should be preferred.

By comparing results obtained using different tip geometries and contact mechanics models, it can be seen that with the increase of radius used in the contact mechanics model, the obtained YM is significantly lower. Larger tips denote larger contact area and interaction volume; thus, the measured area is more averaged. Thus, it was possible to study the elastic properties of spheroids at the multicellular level, while using pyramidal tips allowed us to extract values corresponding to individual elements of the system (i.e., single cells) in the spheroid outer layers. This comparison revealed that using different contact geometries in the fitting procedure results in significantly different YM values, highlighting the importance of a precise experiment design and choice of the AFM probe for measurements.

Since the nature of the cells is linked to their internal structure, i.e., to all components constituting the cell, some of them will have a minor effect on the overall mechanics of the cell, and some will be dominant. The overall conclusion is that, regardless of the cantilever type, the most rigid spheroids were formed from lung fibroblasts, whose cytoskeleton was characterized by F-actin filaments located over the whole cell body. Spheroids composed of rounded cells with actin filaments located at their periphery were more compliant. Moreover, the choice of cantilever type is strongly bound to the relative difference between spheroid types. The difference between the mechanics of lung spheroids formed by spindle-like and round cells was the largest for cantilevers with small CPs, while an analogous large difference for spherical cells forming both lung and colon spheroids was observed when cantilevers with pyramidal tips were used. Using tipless or cantilevers with large CPs flattens the difference between different spheroid types. Our results suggest a dominant contribution of the actin cytoskeleton, as the AFM measurements primarily probe the outer proliferative cell layer rather than deeper intercellular regions of the spheroid. Therefore, even at large indentation depths, the measured mechanical response is expected to remain primarily governed by the actin filaments..

## Supplementary Information

Below is the link to the electronic supplementary material.

Supplementary Material 1
