# Supplementary material for: Indenting multicellular spheroids with various tip geometries
Source: Eur Biophys J. 2026 Apr 6;55(2):319–30. doi: 10.1007/s00249-026-01838-3 (PMC13109145; doi:10.1007/s00249-026-01838-3)
Supplement: Supplementary file 1 — Supplementary Material 1 [file 249_2026_1838_MOESM1_ESM.pdf]

# Supplementary Information

# Indenting multi-cellular spheroids with various tip geometries

Kajangi Gnanachandran[1,2,*,†], Ewelina Lorenc[3,4,*,†], Alessandro Podestà[3,4,°], Małgorzata Lekka[1,°]

[1]Department of Biophysical Microstructures, Institute of Nuclear Physics, Polish Academy of Sciences, Kraków, PL-31342, Poland

[2]Vascular Biology Research Group, Department of Medical Biology, University of Tromsø – The Arctic University of Norway, Tromsø, Norway

[3]Dipartimento di Fisica "Aldo Pontremoli", Università degli Studi di Milano, via G. Celoria 16, 20133, Milano, Italy.

[4]CIMaINa, Università degli Studi di Milano, via G. Celoria 16, 20133, Milano, Italy.

*Corresponding author(s):

E-mail(s): g.kajangi@gmail.com, lorenc.ewelina@gmail.com

[†]These authors contributed equally to this work.

° Co-last authors.

**SUPPLEMENTARY GRAPHS**

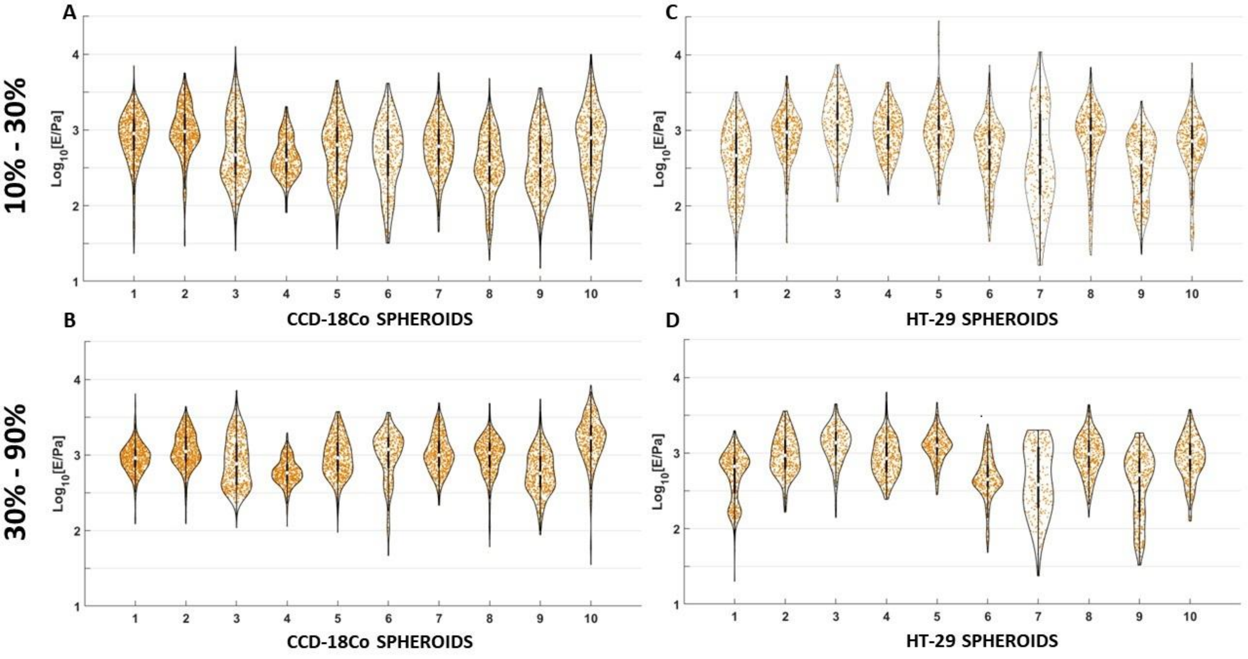


*Supplementary Figure S1:* Violin plots presenting median YM values of each spheroid and distribution of YM obtained from single force-indentation curves (FCs). CCD-18Co measured with indentation of 10%-30% (A); CCD-18Co measured with indentation of 30%-90% (B); HT-29 measured with indentation of 10%-30% (C); HT-29 measured with indentation of 30%-90% (D)

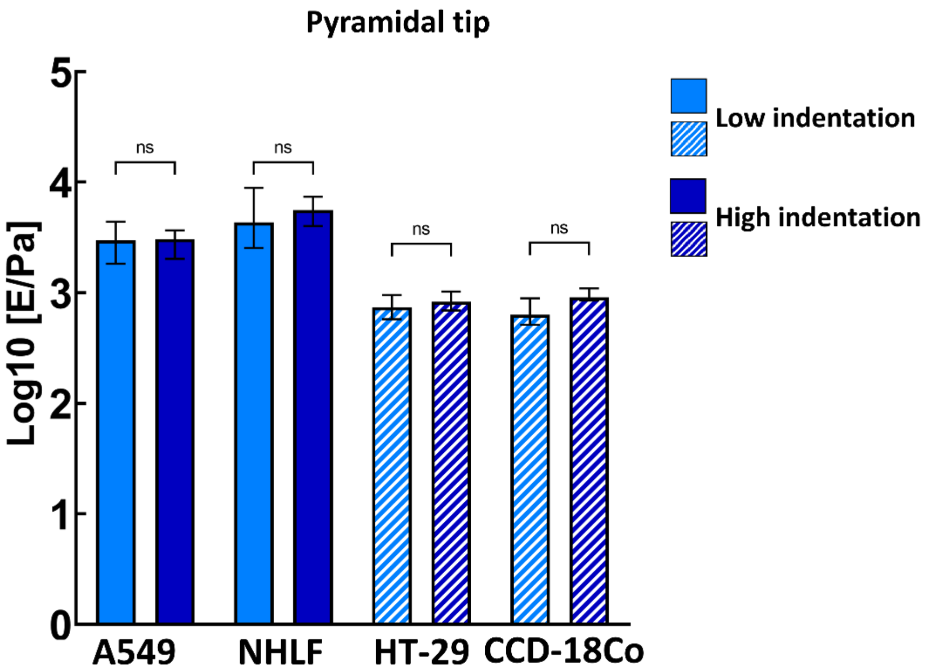


*Supplementary Figure S2:* Comparison of two indentation ranges applied in measurements with pyramidal tip (**** p < 0.0001, ns - not statistically significant). Error bars represent 95% CI of the median.

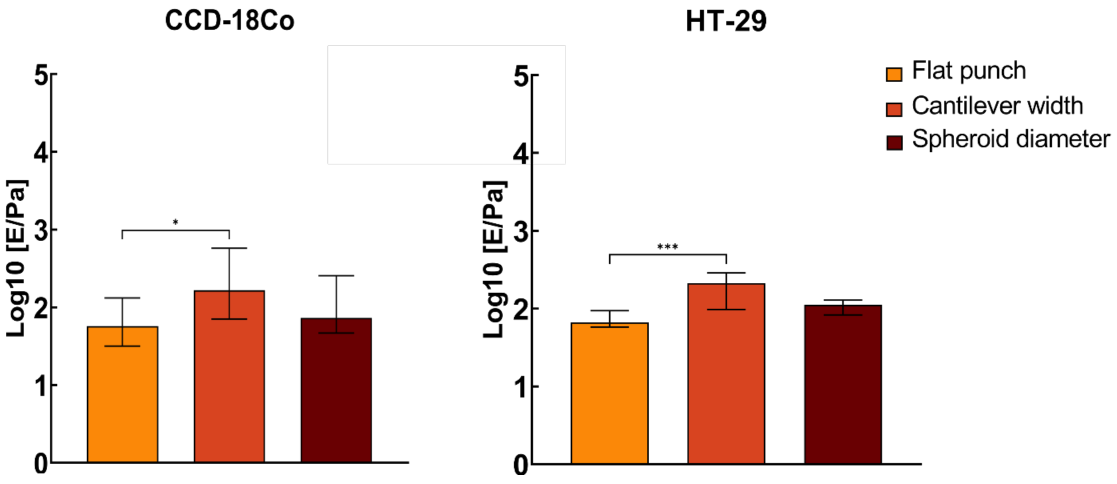


*Supplementary Figure S3*: Results of measurements with tipless cantilever analysed using three different approaches in contact mechanics (**** p < 0.0001, ns - not statistically significant). Error bars represent 95% CI of the median.

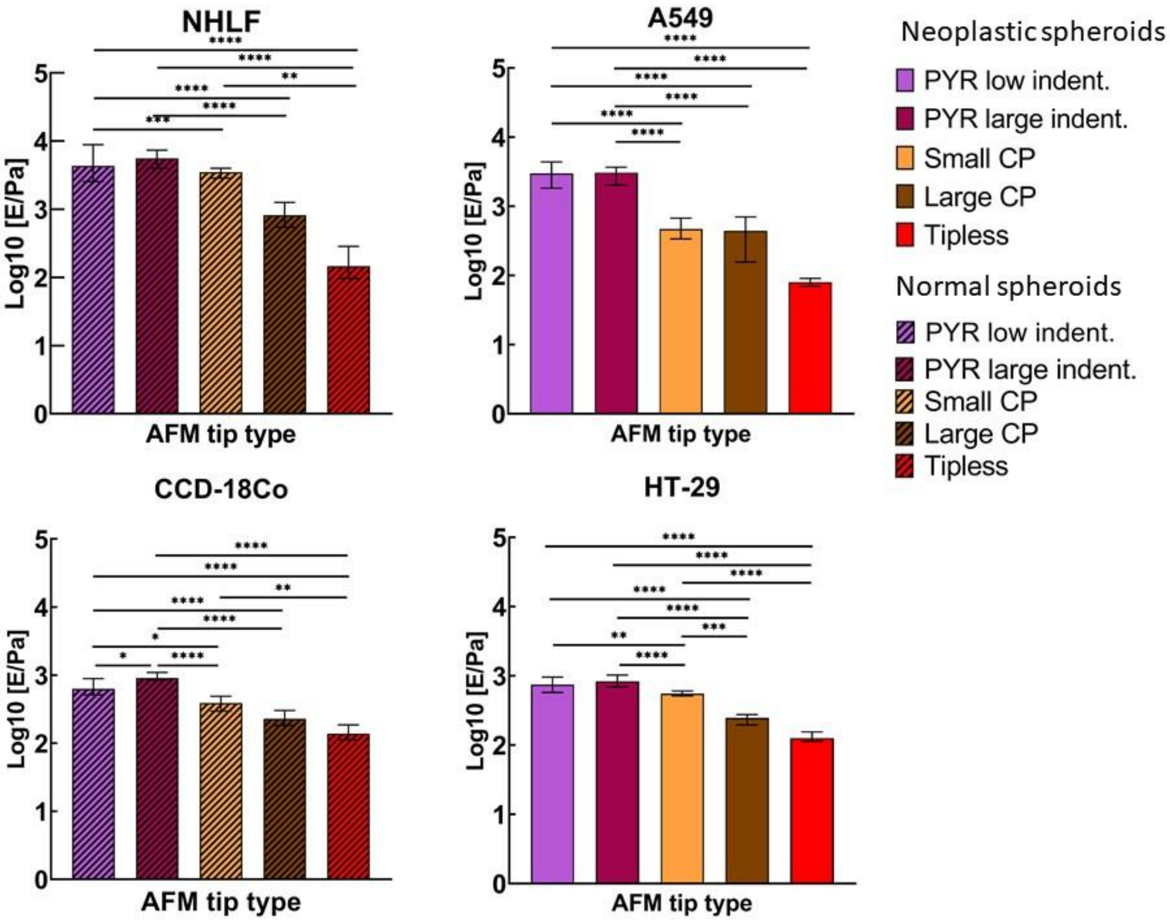


*Supplementary Figure S4:* The comparison between YM median values measured with different AFM tip geometries within each spheroid type. The graphs show a median with a 95% CI of log values (median of the medians). Ordinary ANOVA test with Turkey's multiple comparison test was performed to verify the statistical significance of differences. Error bars represent 95% CI of the median.

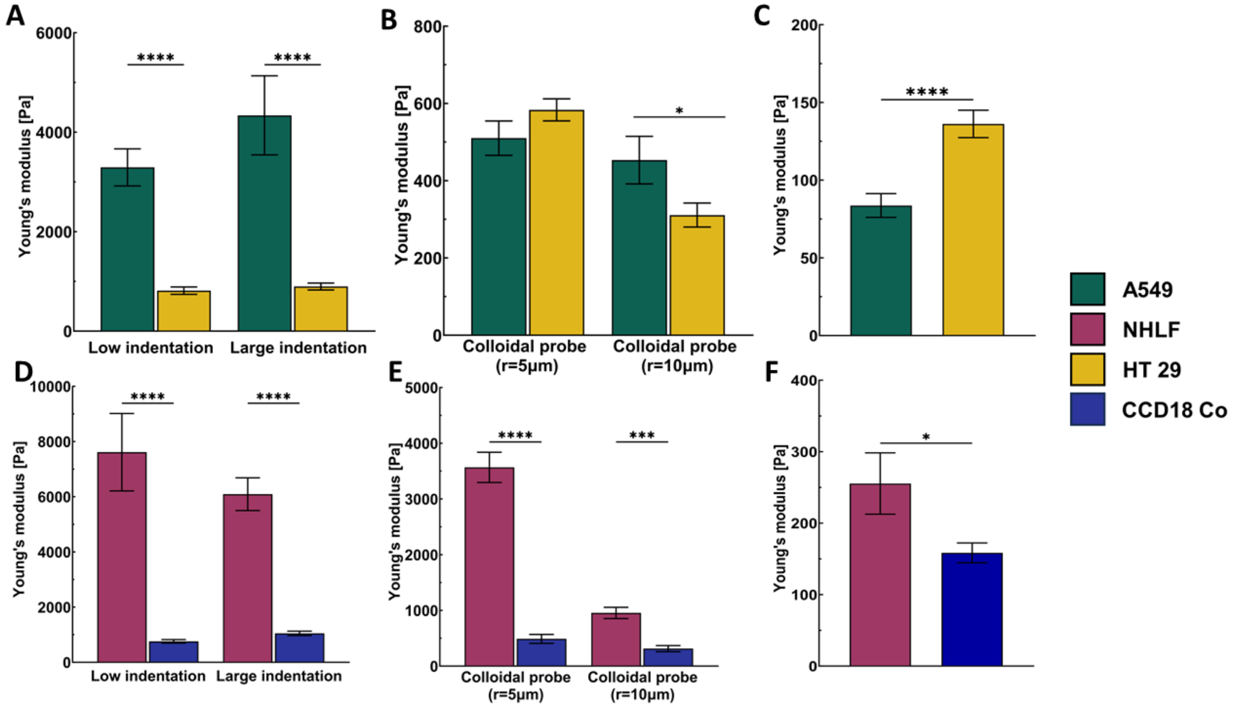


*Supplementary Figure S5:* Comparison of YM values between cancer spheroids (A-C) and between fibroblast-derived spheroids (D-F). The measurements were done with MLCT cantilevers at low and large indentations (A,D), spherical probes (B,E), and tipless cantilevers (C,F). Each bar represents the mean value ± standard error of the mean. (**** $p < 0.0001$, ns - not statistically significant).
